# Pigment Epithelium-Derived Factor Induces Endothelial Barrier Dysfunction via p38/MAPK Phosphorylation

**DOI:** 10.1155/2015/791825

**Published:** 2015-10-04

**Authors:** Ting He, Liping Zhao, Dongxia Zhang, Qiong Zhang, Jiezhi Jia, Jiongyu Hu, Yuesheng Huang

**Affiliations:** ^1^Institute of Burn Research, Southwest Hospital, Third Military Medical University, State Key Laboratory of Trauma, Burns and Combined Injury, Chongqing Key Laboratory for Diseases Proteomics, Chongqing 400038, China; ^2^Department of Endocrinology, Southwest Hospital, Third Military Medical University, Chongqing 400038, China

## Abstract

Endothelial barrier dysfunction, which is a serious problem that occurs in various inflammatory conditions, permits extravasation of serum components into the surrounding tissues, leading to edema formation and organ failure. Pigment epithelium-derived factor (PEDF), which is a major endogenous antagonist, has been implicated in diverse biological process, but its role in endothelial barrier dysfunction has not been defined. To assess the role of PEDF in the vasculature, we evaluated the effects of exogenous PEDF using human umbilical vein endothelial cells (HUVECs) *in vitro*. Our results demonstrated that exogenous PEDF activated p38/MAPK signalling pathway in a dose- and time-dependent manner and induced vascular hyperpermeability as measured by the markedly increased FITC-dextran leakage and the decreased transendothelial electrical resistance (TER) across the monolayer cells, which was accompanied by microtubules (MTs) disassembly and F-actin rearrangement. However, the aforementioned alterations can be arrested by the application of low concentration of p38/MAPK inhibitor SB203580. These results reveal a novel role for PEDF as a potential vasoactive substance in inducing hyperpermeability. Furthermore, our results suggest that PEDF and p38/MAPK may serve as therapeutic targets for maintaining vascular integrity.

## 1. Introduction

The endothelial monolayer that lines all blood vessels functions to control the influx and efflux of materials between the vessel lumen and the interstitium [1]. Loss of endothelial barrier function can lead to increased endothelial permeability, which results in an anarchical movement of fluid, solutes, and cells outside the vasculature and into the surrounding tissues [2], thereby contributing to various diseases such as cancer [3], atherosclerosis [4], lung injury [5], kidney injury [6], stroke, and many other diseases [7]. The current lack of endothelial barrier-promoting therapeutics reflects the historic lack of knowledge of the mechanisms promoting endothelial barrier function. Thus, a better understanding of the molecular mechanisms regulating endothelial barrier function is required for developing new therapies for these diseases.

Pigment epithelium-derived factor (PEDF), a glycoprotein that belongs to the superfamily of serine protease inhibitors, was first purified from the conditioned media of human retinal pigment epithelial cells [8]. PEDF has been implicated in diverse biological process, such as neurogenesis, neuroprotection, antiangiogenesis, stem cell renewal, apoptosis, and inflammation [9]. PEDF is a major antagonist of vascular endothelial growth factor (VEGF) and can effectively inhibit VEGF-driven angiogenesis and vascular permeability by regulating the proteolysis of VEGF receptors [10]. However, recent reports have raised the possibility that PEDF may act as an important endogenous vasoactive substance. Indeed, PEDF has a synergistic action with VEGF by coculture in endothelial cells, and this corresponds to increased mitogen-activated protein kinase (MAPK) activation [11]. Moreover, PEDF activates peroxisome proliferator-activated receptor-*γ* (PPAR-*γ*), which in turn leads to the overexpression of p53 and apoptosis of endothelial cells [12]. Previous studies have shown that a high dose of PEDF increases VEGF mRNA levels in granulosa cells and is not effective at reducing ovarian hyperstimulation syndrome-induced leakage [13]. However, the precise mechanisms underlying the effects of PEDF in the vasculature have not been clarified.

The aim of the present study was to investigate the role of PEDF alone in vascular permeability and to identify the molecular mechanism underlying its effect. We found that exogenous PEDF increased paracellular permeability due to microtubules (MTs) disassembly and F-actin rearrangement by activating the p38/MAPK pathway. Thus, PEDF may play a detrimental role in the maintenance of vascular integrity and may provide a new potential therapeutic target for ameliorating hyperpermeability.

## 2. Materials and Methods

### 2.1. Cell Culture and Reagents

Human umbilical vein endothelial cells (HUVECs) were cultured at 37°C in 5% CO_2_ in endothelial cell medium (ECM, HyClone) containing 10% fetal bovine serum (FBS, Gibco). The culture medium was replaced every 2 to 3 days. After reaching 70% to 80% confluence, the cells were harvested with 0.25% trypsin (ScienCell) and passaged at a ratio of 1 : 3.

Recombinant human SERPINF1/PEDF protein (Sino Biological Inc.) was added and incubated at 37°C at indicated time points. The p38/MAPK inhibitor SB203580 (Calbiochem) was added and incubated at 37°C for 1 h before the PEDF treatment under serum-free conditions.

### 2.2. Western Blotting (WB)

Cells were washed 3 times with PBS (4°C) and lysed in RIPA buffer (ProteinSimple). Cell lysates were heated to 95°C in SDS loading buffer, and the protein concentration was determined using the RCDC protein detection assay (Bio-Rad). Proteins separated by SDS-PAGE were transferred onto polyvinylidene difluoride (PVDF) membranes (Millipore), blocked in 5% skim milk, and incubated with primary and secondary antibodies. p38/MAPK (1 : 1000, Cell Signaling Technology), p-p38/MAPK (1 : 1000, Thr180/182, Cell Signaling Technology), and GAPDH-HRP (1 : 5000, Proteintech) antibodies were used. Bands were visualized by chemiluminescence using the ChemiDocTM XRC^+^ Imaging System (Bio-Rad) and enhanced chemiluminescence (ECL) detection reagents (GE Healthcare).

### 2.3. Immunofluorescence Staining

Immunocytochemistry was performed using a standard procedure. In brief, cells were grown on glass coverslips and cultured as described above. The cells were washed 3 times with PBS (37°C), fixed in 4% paraformaldehyde for 20 min, and permeabilised with 0.1% TritonX-100 for 15 min.Cells were incubated for 1 h in 5% normal goat serum, followed by immunofluorescence staining. To obtain the MTs structure, rabbit anti-*α*-tubulin primary antibodies (1 : 50, Proteintech) were diluted with PBS, and the coverslips were incubated at 4°C overnight. The coverslips were washed in PBS and then incubated with goat anti-rabbit secondary antibodies conjugated to cyanine 3 (Cy3, 1 : 100, Proteintech) for 1 h at 37°C. To obtain the F-actin structure, rhodamine phalloidin (1 : 50, Sigma-Aldrich) was diluted with PBS, and the coverslips were incubated at 4°C overnight. The cells were imaged using confocal microscopy (Leica).

### 2.4. Assessment of Cell Permeability

Transwell tracer experiments were performed using the 24-well Transwellsystem(0.4 *μ*mpore size, 6.5 mm diameter, Costar). HUVECs (1 × 10^5^) were plated in Transwell chambers, grown for 3 days, and serum-starved for 4 h. To assess endothelial permeability, the paracellular flux and transendothelial electrical resistance (TER) were measured after stimulation. To measure paracellular flux, the media from the basolateral domain were collected at the indicated times after FITC-dextran (40 KDa; Sigma-Aldrich) addition for 1 h. TERwasmeasured using a Millicell ERS-2 voltohmmeter (Millicell). At the indicated times, resistance (Ω) was obtained from each insert and multiplied by the membrane area (*Ω* × cm^2^) to obtain values of TER. The resistance value of an empty culture insert (no cells) was subtracted as background.

### 2.5. Statistical Analysis

Statistical analysis between groups was performed using a two-tailed one-way analysis of variance (ANOVA) followed by Tukey's post hoc analysis. Statistical analyses were performed using Statistical Product and Service Solutions (SPSS 19.0, SPSS Inc.). Differences were considered statistically significant at a *P* value < 0.05.

## 3. Results

### 3.1. PEDF Activated p38/MAPK Signalling Pathway in a Dose- and Time-Dependent Manner

**Figure 1:**
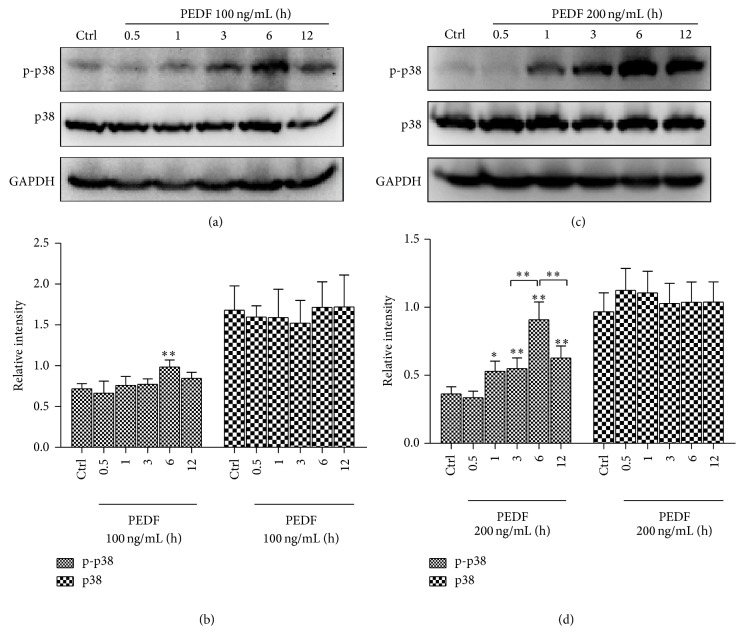
PEDF activated p38/MAPK signalling pathway in dose- and time-dependent manner. Representative blots and data summary of phospho-p38 (p-p38) and p38 in HUVECs under PEDF (100/200 ng/mL) treatment at indicated time points. Representative western blots are shown for the two groups. Graph represents the mean ± SD (*n* = 3 in each group) of the relative integrated signals. ^*^ *P* < 0.05, ^**^ *P* < 0.01.

We first investigated whether PEDF activated p38/MAPK signalling pathway in endothelial cells. Different concentrations of exogenous PEDF were adopted. Phosphorylated (Thr180/Tyr182) p38/MAPK was tested by immunoblotting with a phosphor-specific p38/MAPK antibody. p38/MAPK phosphorylation was significantly elevated at 6 h after PEDF (100 ng/mL) treatment, with a 1.36-fold increase compared with control group (Figures 1(a) and 1(b)). Phosphorylation of p38/MAPK was significantly elevated by PEDF (200 ng/mL) stimulation from 1 h to 12 h and peaked at 6 h, with a 2.49-fold increase compared with control group (Figures 1(c) and 1(d)). These results demonstrated that PEDF activated p38/MAPK in a dose- and time-dependent manner in endothelial cells.

### 3.2. Low Concentration of SB203580 Abolished the PEDF-Induced p38/MAPK Phosphorylation

**Figure 2:**
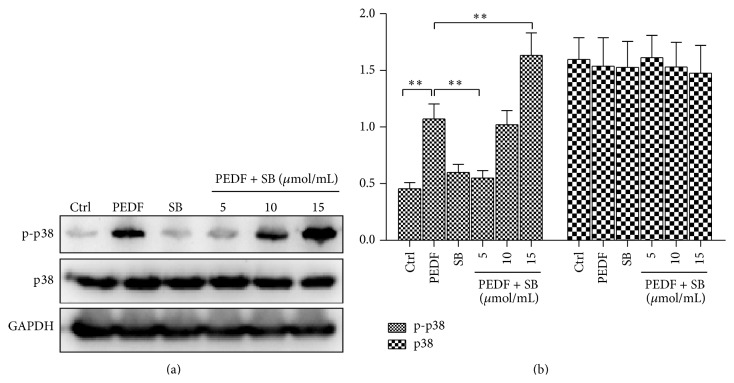
Low concentration of SB203580 abolished the PEDF-induced p38/MAPK phosphorylation. Representative blots and data summary of phospho-p38 (p-p38) and p38 in HUVECs under PEDF (200 ng/mL), SB203580 (5 *μ*mol/mL), and PEDF (200 ng/mL) + SB203580 (5/10/15 *μ*mol/mL) treatment at 6 h. Representative western blots are shown. Graph represents the mean ± SD (*n* = 3 in each group) of the relative integrated signals. ^*^ *P* < 0.05, ^**^ *P* < 0.01.

The clear activation of p38/MAPK described above led us to select the 6 h time point after PEDF (200 ng/mL) treatment for subsequent experiments. We next validated the optimal concentration of p38/MAPK inhibitor SB203580, which could abolish the p38/MAPK phosphorylation. Cells were treated with different concentrations of SB203580 (5/10/15 *μ*M), and only 5 *μ*M SB203580 could abolish the p38/MAPK phosphorylation induced by PEDF (200 ng/mL), with a 1.95-fold decrease compared with PEDF group (Figure 2). Interestingly, when SB203580 was used at 10 *μ*M or 15 *μ*M, it failed to decrease the p38/MAPK phosphorylation induced by PEDF, and the p38/MAPK was significantly phosphorylated at 15 *μ*M SB203580 treatment, with a 1.53-fold increase versus PEDF alone (Figure 2).

### 3.3. Role of p38/MAPK Activation in PEDF-Induced Endothelial Barrier Dysfunction

**Figure 3:**
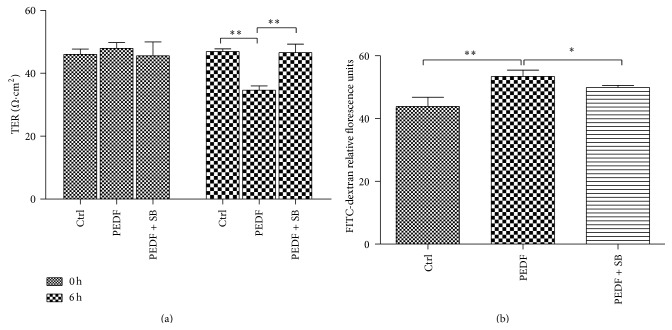
Role of p38/MAPK activation in PEDF-induced endothelial barrier dysfunction. (a) Temporal changes in TER were measured across a microvascular endothelial monolayer grown on a Transwell insert treated with vehicle (unstimulated), PEDF (200 ng/mL), and PEDF (200 ng/mL) + SB203580 (5 *μ*mol/mL). Analyses were performed in triplicate, and the data are shown as the mean ± SD. (b) Endothelial cells were incubated under the conditions described in Figure 3(a). Paracellular permeability was measured following the addition of FITC-dextran (40 kDa) for 1 h. Analyses were performed in triplicate, and the data are shown as the mean ± SD. ^*^ *P* < 0.05, ^**^ *P* < 0.01.

Next we observed the effect of p38/MAPK on endothelial barrier function in HUVECs. We found that SB203580 (5 *μ*M) largely abolished the PEDF-induced paracellular hyperpermeability, as indicated by increased TER (1.35-fold increase compared with the value of PEDF group) across endothelial cell monolayers and decreased FITC-dextran leakage (1.06-fold decrease compared with the value of PEDF group) (Figure 3). These results demonstrated that p38/MAPK signalling pathway played a pivotal role in PEDF-induced vascular hyperpermeability.

### 3.4. MTs Disassembly and F-Actin Rearrangement Were Involved in PEDF-Induced Endothelial Barrier Dysfunction

**Figure 4:**
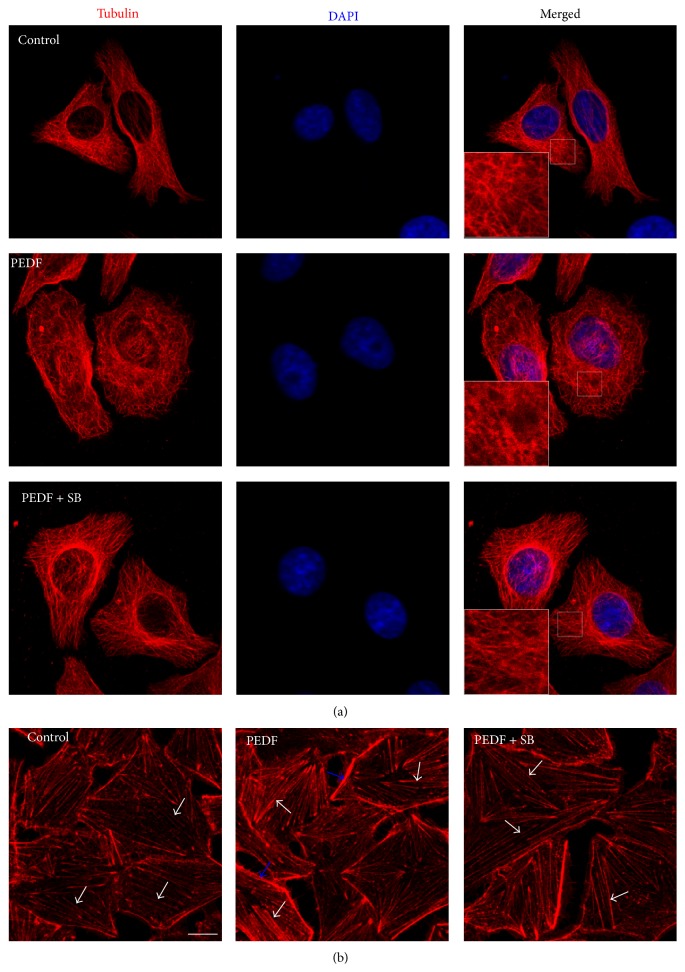
MTs disassembly and F-actin rearrangement were involved in PEDF-induced endothelial barrier dysfunction. (a) Endothelial cells were incubated under the conditions described in Figure 3(a). All the cells were stained for a-tubulin (red) and the nucleus was stained for DAPI (blue). Representative images show the MTs organization. The boxed areas are shown at higher magnification in the inserts to illustrate details of MTs. Scale bars = 10 *μ*m. (b) Endothelial cells were stained for F-actin (red). White and blue arrows indicate central and peripheral F-actin fibers in cells, respectively. Scale bars = 10 *μ*m.

In the morphological studies, the HUVECs subjected to PEDF showed clear signs of MTs disruption and modification. In control endothelial cells, the MTs are organized into a faint, uniformly distributed lattice network, whereas the PEDF (200 ng/mL) challenge caused a less regular organization and some breakages that changed the MTs appearance (Figure 4(a)). Concomitant with the prevention of hyperpermeability, SB203580 preserved the MTs network in the PEDF-challenged cells (Figure 4(a)). Staining of F-actin revealed a profound change in cell morphology. In particular, cells in the control group demonstrated long thin F-actin fibers in the central portion of cells (Figure 4(b), white arrow). However, in cells stimulated with PEDF, the central F-actin bundles (white arrow) were obvious and thicker, and dense wider peripheral F-actin (blue arrow) occurred (Figure 4(b)). However, SB203580 preserved the F-actin rearrangement in the PEDF-challenged cells, and long thin F-actin fibers (white arrow) occurred in the central portion of cells (Figure 4(b)). Taken together, these results provided clear evidence that PEDF induced endothelial barrier dysfunction by MTs disassembly and F-actin rearrangement.

## 4. Discussion

In the present study, we identified that PEDF induced endothelial barrier dysfunction in HUVECs through activating the p38/MAPK signalling pathway, which led to MTs disassembly, F-actin rearrangement, and a parallel increase in vascular permeability, as indicated by the markedly increased FITC-dextran leakage and the decreased TER across the monolayer cells.

Although the endothelium is an extremely thin single-cell layer, it constitutes a selective barrier between blood and tissue and performs exceedingly well in preventing blood fluids from leaking into the surrounding tissues [14]. However, specific pathological conditions can affect the monolayer cells, compromising the integrity of the endothelial barrier. Vascular leakage is a hallmark of many diseases and no specialized therapies are available to prevent it or reduce it [15].

PEDF, an endogenously produced protein that belongs to the superfamily of serine protease inhibitors, is one of the most potent inhibitors of angiogenesis [16]. It is well known that PEDF can inhibit VEGF-induced vascular hyperpermeability [17]. But the elevated PEDF in diabetic patients is found to be associated with microvascular complications and poor vascular health [18]. The conclusions from previous studies suggest that PEDF exerts several important but contradictory influences on the vascular endothelial barrier [10, 12, 19]. In the current study, we found that exogenous PEDF led to an increase in paracellular permeability in HUVECs, which was consistent with a previous study that PEDF when used alone at high concentrations did not block VEGF but caused an increase in paracellular permeability inversely [20].

Therefore, PEDF alone may act as a potent propermeability molecule.

An important question arises as to which signalling pathway in endothelial cells is responsible for the PEDF-induced hyperpermeability. Indeed, PEDF was shown to activate MAPK pathways to regulate endothelial proapoptotic and antimigratory activities [19, 21]. MAPKs are proline-directed serine and threonine protein kinases that mediate a wide variety of cellular behaviors in response to extracellular stimuli [22]. One of the main subgroups, the p38/MAP kinases, has been implicated in a wide range of complex biologic processes [23]. In the endothelium, the p38/MAPK pathway plays a role in regulating cell proliferation, migration, and permeability, during various pathological insults [24, 25]. An activation of p38/MAPK, accompanied by rearrangement of F-actin and phosphorylation of L-caldesmon and MAP4, does harm to the vascular endothelial barrier function [26, 27]. In this study, we focused on the role of the p38/MAPK signalling pathway and identified an activation of this pathway after PEDF stimulation in a dose- and time-dependent manner. Moreover, low concentration of p38/MAPK inhibitor SB203580 abolished the PEDF-induced p38/MAPK phosphorylation. It is interesting to find that the use of high concentrations of SB203580 increases PEDF action. SB203580 exerts its inhibitory effect by binding to the ATP binding pocket of p38/MAPK, thus inhibiting its ability to undergo autophosphorylation but not affecting the capacity of p38/MAPK to be phosphorylated by upstream MAPKK and MKK3/6 [28]. Therefore, it is possible to consider a compensatory effect of other signalling pathways when SB203580 is used at high concentrations. Further studies are needed to determine the signalling pathways involved.

Disruption of the cytoskeleton (MTs, microfilaments, and intermediate filaments) and cell junctions plays a pivotal role in increasing vascular permeability [29]. MTs dynamics in vascular endothelium are modulated by vasoactive mediators and are critically involved in the control of endothelial cell permeability via Rho GTPase-dependent crosstalk with the actin cytoskeleton [30]. In the present study, we tested the hypothesis that p38/MAPK activation was responsible for the MTs disassembly, F-actin rearrangement, and the hyperpermeability in HUVECs. In contrast, SB203580, the p38/MAPK inhibitor, promoted MTs and F-actin remodeling and reduced the hyperpermeability of HUVECs. However, further studies are needed to determine the intermediate effectors linking PEDF and p38/MAPK activation.

## 5. Conclusions

In summary, we conclude that p38/MAPK activation plays a critical role in the PEDF-induced MTs disassembly, F-actin rearrangement, and the subsequent vascular hyperpermeability. Our data suggest that PEDF and p38/MAPK may be useful targets for the specific modulation of vascular hyperpermeability.
